# Type I Toxin-Antitoxin Systems in Clostridia

**DOI:** 10.3390/toxins11050253

**Published:** 2019-05-06

**Authors:** Olga Soutourina

**Affiliations:** Institute for Integrative Biology of the Cell (I2BC), CEA, CNRS, Univ. Paris-Sud, Université Paris-Saclay, 91198 Gif-sur-Yvette CEDEX, France; olga.soutourina@i2bc.paris-saclay.fr; Tel.: +33-1-69-82-62-06

**Keywords:** toxin-antitoxin systems, type I, RNA antitoxins, *Clostridium difficile*, prophages

## Abstract

Type I toxin-antitoxin (TA) modules are abundant in both bacterial plasmids and chromosomes and usually encode a small hydrophobic toxic protein and an antisense RNA acting as an antitoxin. The RNA antitoxin neutralizes toxin mRNA by inhibiting its translation and/or promoting its degradation. This review summarizes our current knowledge of the type I TA modules identified in Clostridia species focusing on the recent findings in the human pathogen *Clostridium difficile*. More than ten functional type I TA modules have been identified in the genome of this emerging enteropathogen that could potentially contribute to its fitness and success inside the host. Despite the absence of sequence homology, the comparison of these newly identified type I TA modules with previously studied systems in other Gram-positive bacteria, i.e., *Bacillus subtilis* and *Staphylococcus aureus,* revealed some important common traits. These include the conservation of characteristic sequence features for small hydrophobic toxic proteins, the localization of several type I TA within prophage or prophage-like regions and strong connections with stress response. Potential functions in the stabilization of genome regions, adaptations to stress conditions and interactions with CRISPR-Cas defence system, as well as promising applications of TA for genome-editing and antimicrobial developments are discussed.

## 1. Introduction

Toxin-antitoxin loci (TA) are widespread in prokaryotes and encode a stable toxin component and an unstable antitoxin to repress the function or expression of the toxin [1]. The toxin is always of a protein nature while the antitoxin can be a protein or a non-coding RNA (ncRNA). The overexpression of the toxin inhibits growth or induces cell death, while the antitoxin protects cells from the toxin’s action. TA loci were initially discovered on plasmids where they confer stability of maintenance through post-segregation killing and now are well known as a part of mobilome [2]. TA systems are also abundant on bacterial and archaeal chromosomes but their function remains largely unclear. The biological roles that are suggested for these enigmatic chromosomal TA modules include prophage maintenance, preventing of phage infection, stress response, and persister formation [1,3,4,5].

TA families are divided into six types depending on the nature and action of the antitoxin [5]. In the most-studied type II TA modules, the protein antitoxin binds directly to the toxin and neutralizes it. In type I and type III TA systems, the antitoxins are ncRNAs [6]. In type III TA systems, the antitoxin RNA binds to the toxin protein to sequester it in an inactive form [7]. In type I TA systems, the antitoxin is a small antisense RNA that base-pairs with the toxin-encoding mRNA, altering its stability and/or translation [6,8]. Type I toxins are usually small hydrophobic proteins less than 60 amino acids in length containing a potential transmembrane domain and charged amino acids at the C-terminus [9]. The mechanism of action similar to phage holins has been suggested in most cases by inducing pores in cell membranes and impairing ATP synthesis [6]. Consequently, replication, transcription, and translation may be inhibited, which could lead to growth stasis, persistence, or even cell death.

In this review, we will focus on the recently described type I TA modules in the human pathogen *Clostridium difficile*. *C. difficile* is a Gram-positive, strictly anaerobic spore-forming bacterium that became one of the major nosocomial enteropathogens in the industrial countries [10,11]. *C. difficile*-associated diarrhoea is currently the most frequently occurring nosocomial diarrhoea worldwide. For the last approximately ten years, the proportion of severe infection forms rose due to the emergence of a hypervirulent and epidemic 027/NAP1/BI lineage [12]. Two major risk factors to contracting *C. difficile* infection are age and antibiotic exposure. Indeed, antibiotic therapy causes alterations in colonic microflora known as dysbiosis that facilitates implantation or development of *C. difficile,* after contamination by spores or in the presence of endogenous *C. difficile* in healthy carriers [11,13]. This pathogen synthesizes two major toxins, TcdA and TcdB, inducing the alterations in the cytoskeleton of intestinal epithelial cells [14,15]. This leads to intestinal cell lysis and inflammation resulting in diarrhoea, pseudomembranous colitis, and even death. Other virulence factors have been identified that contribute to the colonization of the host [16]. Despite antibiotic treatments, one of the most challenging traits of *C. difficile* infection is the high rate of recurrent infections that can even increase after a second and third recurrence [17]. The ability to form highly resistant spores during an infection cycle is essential for the relapse of *C. difficile* infection [18]. Nevertheless, additional mechanisms, including TA systems, could contribute to the fitness and success of this pathogen inside the host.

## 2. Type I TA Systems in *Clostridium difficile*

TA modules are generally abundant in all prokaryotes but have not been largely studied in Clostridia. As TA systems have been potentially implicated in the persistence after antibiotic treatment and stress responses, some recent efforts have been made to search for TA modules in Clostridial pathogens and commensal species. A recent genomics study revealed the presence of TA systems in the *C. tetani* genome [19]. Exhaustive sequence homology searches for putative type III TA systems belonging to three major families identified a potential *toxIN* locus in Clostridial species, i.e., *Clostridium botulinum*, *Clostridium cellulovorans*, *Clostridium nexile, Clostridium phage D-1873*, and *Clostridium sp.;* a potential *cptIN* locus in *Clostridium hiranonis* and *Clostridium sp.;* and a potential *tenpIN* locus in *C. hiranonis* awaiting further characterization [20]. A counterpart of a well-studied MazEF type II TA system has been identified in *C. difficile* with a MazF-cd toxin that exhibits selective mRNA cleavage [21]. The in silico prediction of three additional putative type II TA systems has been recently described in *C. difficile* and their possible implication in recurrent infections together with sporulation and biofilm formation has been discussed [22].

The large in silico search for homologs to known type I toxins in bacterial genomes could not find such proteins in the majority of Clostridial species with the exception of TxpA-like and Ldr/Fst-like sequences in *Clostridium bolteae* and a Fst-like sequence in *Clostridium asparagiforme* [9]. Nevertheless, the in silico search for characteristic sequence features with clusters of charged and bulky amino acids at the C-termini of short proteins containing predicted transmembrane regions resulted in a number of candidates for novel type I toxins in bacteria [9]. In Clostridial species, several potential type I toxin candidates have been predicted by this analysis in *Clostridium acetobutylicum* (4 proteins), *Clostridium beijerinckii* (3 proteins), *C. botulinum* (from 4 up to 14 proteins depending on the strain), *C. difficile* (9 proteins), *Clostridium kluyveri* (5 proteins), *Clostridium novyi* (1 protein), *Clostridium perfringens* (from 3 to 5 proteins depending on the strain), and *Clostridium thermocellum* (1 protein) awaiting further studies.

**Figure 1:**
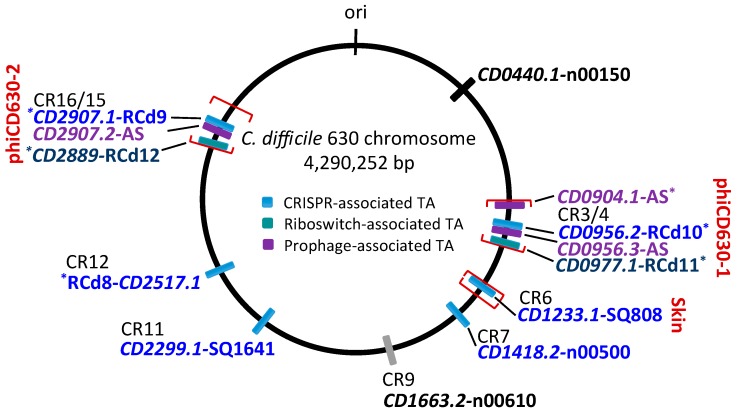
Schematic genomic map of type I TA loci in *C. difficile* strain 630. The location of type I TA modules is shown in blue for CRISPR-associated loci, in green for c-di-GMP-responsive riboswitch-associated loci, and in purple for additional prophage-associated TA loci encoding 34–35 amino acid proteins. The prophage position is indicated using square brackets for phiCD630-1, phiCD630-2, and *skin* element. * indicates TA modules with detailed characterization. CRISPR arrays (CR) are numbered according to CRISPRdb (https://crispr.i2bc.paris-saclay.fr/crispr/) and previous publications [23,26,27,28]. The *CD1663.2*-n00610 locus associated with CRISPR 9 array but encoding a small protein with a divergent sequence is indicated in grey. “ori” indicates the origin of replication.

**Figure 2:**
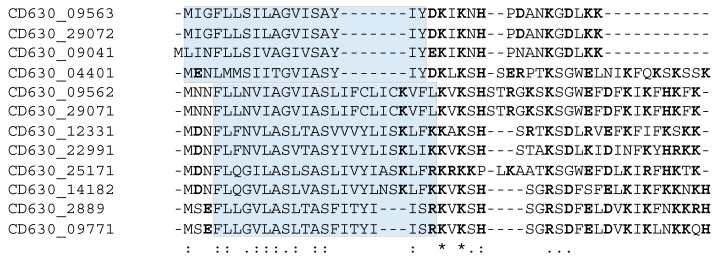
Alignment of toxins from *C. difficile* type I TA modules using MUSCLE 3.8. * indicates conserved amino acids, “:” indicates strongly similar amino acids, “.” indicates weakly similar amino acids. The transmembrane domains predicted using TMHMM (http://www.cbs.dtu.dk/services/TMHMM/) and/or TMpred (https://embnet.vital-it.ch/software/TMPRED_form.html) programs are shaded and charged amino acids are shown in bold.

Our current knowledge of the type I TA systems in Clostridia is still limited and restricted to experimentally validated TA modules in *C. difficile* (Table 1, Figure 1). Until now, a total of 13 potential type I TA modules could be found in the genome of this pathogen, where seven of them correspond to the in silico predictions of type I toxins [9,23,24]. We have recently provided the first data on the experimental identification of type I TA systems in *C. difficile* genome [23]. In contrast to more easily defined type II TA protein families, the direct search for type I TA components including RNA antitoxins remains difficult. By combining in silico prediction and genome-wide promoter mapping, we have recently identified more than 200 ncRNAs in *C. difficile* [25,26]. The analysis of these deep-sequencing transcriptomic data for the antisense RNAs that overlap the genes encoding small proteins of unknown function allowed us to describe six potential TA loci in the laboratory strain 630∆*erm* [23]. Three of these TA modules, i.e., *CD2517.1*-RCd8, *CD2907.1*-RCd9, and *CD0956.2*-RCd10, were composed of the overlapping toxin and antitoxin genes in convergent orientation and have been characterized in some detail. The newly identified toxin genes were not previously annotated in the *C. difficile* genome and have no sequence homology with known type I toxins from other bacteria. However, these small proteins of 50–53 amino acids in length share the characteristic features of type I toxins (Figure 2). The experimental evidence for their membrane localization and toxic nature has been provided in accordance with the presence of a predicted transmembrane domain in the N-terminal part (Figure 2) [23]. Toxin overexpression in the plasmid led to the growth arrest in *C. difficile* and cognate antitoxin co-expression in *cis* or in *trans* was necessary for *C. difficile* survival. Some mechanistic studies have also been performed to start the elucidation of the antitoxin action on toxin mRNA [23]. Efficient duplex formation between toxin mRNA and antisense antitoxin RNA was demonstrated in vitro. Half-life measurements revealed that, similar to previously reported type I TA modules in other bacteria, these TA loci encode a stable toxin and unstable antitoxin RNAs.

**Figure 3:**
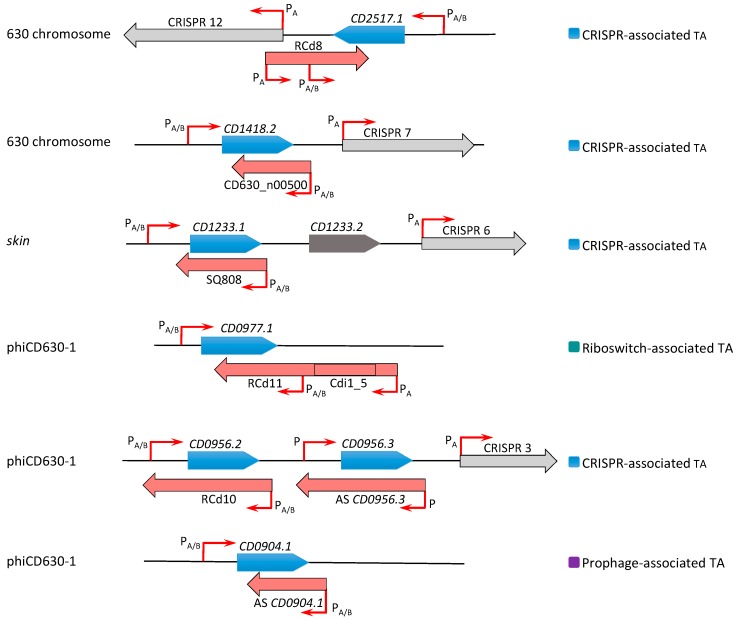
Schematic representation of type I TA regions in the *C. difficile* chromosome. Toxin coding regions are shown as blue arrows, antitoxins are shown in red, and CRISPR arrays in grey. The broken red arrows indicate the position of transcriptional start sites associated with “P_A_” for Sigma A- or “P_A/B_” for Sigma A- and Sigma B-dependent promoters. The specific genomic region including prophage is indicated to the left. On the right, the association with CRISPR arrays or c-di-GMP-dependent riboswitches is shown with the same colour code as in Figure 1.

Interestingly, these TA modules are associated with CRISPR arrays from CRISPR (clustered regularly interspaced short palindromic repeats)-Cas (CRISPR-associated) systems for defence against foreign nucleic acids [23,27,28,29] (Figure 1 and Figure 3). A large analysis of 2500 available *C. difficile* genomes suggested a conservation of this association in the majority of sequenced *C. difficile* strains [23]. In addition, the promoter specific to general stress response Sigma B factor has been identified upstream of both the toxin and antitoxin genes and *cas* operons. We have shown that both the CRISPR-Cas and TA components were downregulated in the *sigB* mutant as compared to the parental strain and induced within biofilms as compared to planktonic cultures [23]. Such co-localization and co-regulation by the general stress response Sigma B factor and biofilm-related factors suggested a possible genomic link between these cell dormancy and adaptive immunity systems in accordance with the recently emerged concept of functional coupling of defence systems in prokaryotes [30]. Two of these functional type I TA pairs (*CD0956.2*-RCd10 and *CD2907.1*-RCd9) are located within the homologous phiCD630-1 and phiCD630-2 prophage regions in the *C. difficile* 630 strain (Figure 1; Figure 3). Another type I TA module *CD1233.1*-SQ808 is located within the *sigK* intervening (*skin*) sequence element, a prophage-like element integrated into the *sigK* gene encoding the late sporulation sigma factor, which is excised from the chromosome during sporulation. This location is reminiscent of the *txpA*-RatA type I TA module in the *Bacillus subtilis skin* element [31], but no sequence homology was observed between these loci.

In the further work, we described the identification of five additional type I TA modules highly conserved within *C. difficile* prophage regions and provided experimental evidence of their possible contribution to these genomic regions’ stability [24]. Two of these TA modules *CD0977.1*-RCd11 and *CD2889*-RCd12, located in the homologous phiCD630-1 and phiCD630-2 prophage regions in the *C. difficile* 630 strain, are composed of the toxic protein of 47 amino acids in length and an antitoxin RNA associated with type I riboswitches responding to c-di-GMP signalling molecule: cdi1_4 and cdi1_5 (Table 1, Figure 3) [26]. Second Sigma A- and Sigma B-dependent promoters have been detected using transcriptional start site mapping and RACE (rapid amplification of cDNA ends) experiments downstream from cdi1_4 and cdi1_5 riboswitches that could efficiently drive the transcription of a short abundant antitoxin sufficient for toxin inactivation (Figure 3) [24]. Three additional type I TA pairs were found within prophage regions in the *C. difficile* strain 630 via a tBlastN homology search using a CD0956.2 type I toxin as a query. These TA modules encode functional type I toxins CD0956.3 and CD0904.1 of 34 and 35 amino acids in length within phiCD630-1 prophage and a 34-amino-acid toxin CD2907.2 within prophage phiCD630-2 [24].

One more potential type I TA module *CD0440.1*-n00150 could be identified using homology searches for other characterized *C. difficile* type I toxins and confirmed using previously reported RNA-seq data [26]. Altogether these recent findings bring the total number of chromosomal type I TA modules to 13, suggesting their functional importance for *C. difficile* (Table 1). The presence of multiple type I TA modules raises the question regarding their functional redundancy and possible crosstalk between different TA loci. Despite extensive homology between several studied TA regions, in most cases only cognate antitoxins were able to counteract the growth inhibition via the corresponding toxins, suggesting the extreme specificity of antitoxin action [23,24]. One exception was observed for almost identical TA pairs from phiCD630-1 and phiCD630-2 prophages. Indeed, we reported the ability of a highly similar RCd12 antitoxin to replace the RCd11 antitoxin carrying only three mismatches for repression of the non-cognate CD0977.1 toxin [24]. Furthermore, no cross-interaction between non-cognate TA pairs was generally found in previous studies of other type I and type II TA modules [32,33,34,35]; however, several examples of non-cognate interactions have been reported for type II TA modules, e.g., VapBC in *Haemophilus influenzae*; RelBE, MazEF, and VapBC in *Mycobacterium tuberculosis* [36,37,38].

The bioinformatics analysis of available *C. difficile* phage genomes indicated that the homologs of type I toxins are widespread in *C. difficile* phages and prophages [24]. These homologous proteins have variable length from 30 to 56 amino acids but carry a conserved hydrophobic N-terminal and lysine-rich charged C-terminal region. Such large distribution of these small proteins within *C. difficile* prophages further emphasizes their functional importance. In addition, an extended search outside *C. difficile* phages revealed the presence of other type I toxin homologs inside plasmids of *C. difficile* and *Paeniclostridium sordellii*, a closely related species [24].

## 3. Comparison with Type I TA Described in Other Gram-Positive Bacteria

Comparison of newly identified type I TA modules in *C. difficile* with previously studied TA systems in other bacteria revealed no sequence homology for small toxin proteins except conservation of their membrane association and the presence of charged amino acids in C-terminal part [9,39,40]. The co-localization of functional type I TA systems with CRISPR arrays that we observed on the *C. difficile* chromosome (Figure 1 and Figure 3) was not previously reported in other bacterial genomes [23]. This would not be relevant for *B. subtilis* with no CRISPR arrays present in its genome [41], but localization of several type I TAs within prophage or prophage-like regions constitutes a common feature between identified *C. difficile* and *B. subtilis* systems (Figure 1 and Figure 3) [6,23,24,31,42,43]. Similarly to *B. subtilis* systems, the role in stabilization of these chromosomal regions could be suggested for TA in *C. difficile* carrying a high proportion of stable mobile genetic elements in its genome [44].

In Gram-positive bacteria, the type I TA systems have been extensively studied in the model bacterium *B. subtilis* and in the human pathogen *Staphylococcus aureus* [6,8,43,45]. Type I TA modules are highly represented in *B. subtilis* chromosome. After the first discovery of the *txpA*-RatA TA module in this bacterium [31], further deep sequencing and in silico analyses have brought the total number of type I TA modules to 14 in *B. subtilis*, which are organized in four families—TxpA/BsrG, BsrH/BsrE, YonT, and YhzE—on the basis of the sequence similarities between type I toxins [43]. In the absence of the sequence similarities between type I toxins identified in *C. difficile* and *B. subtilis*, they still share some common features. This includes their small size from 28 to 60 amino acids in *B. subtilis* and from 34 to 59 amino acids in *C. difficile*, charged amino acid region in C-termini, transmembrane domain, toxicity through overexpression, and finally, location of most of them in prophage regions that could contribute to prophage stability. Eight type I TA modules are located within phiCD630-1 and phiCD630-2 prophages and the *skin* element in the *C. difficile* strain 630 chromosome, while at least five type I TA modules—*bsrG*-SR4/*yonT*-SR6, *bsrE*-SR5, *txpA*-RatA/*bsrH*-AS-*bsrH*—reside in the SPβ, P6 prophages, and *skin* element of *B. subtilis* chromosome, respectively. As observed in *C. difficile* (Figure 1), some *B. subtilis* prophages carry several TA modules, for example, *bsrG*-SR4 and *yonT*-SR6 in the SPβ prophage, and *txpA*-RatA and *bsrH*-AS-*bsrH* in the *skin* element [43]. Detailed molecular and functional characterization has been provided for TxpA/BsrG and YonT families of type I TA modules in *B. subtilis* [31,39,42,46,47,48,49,50,51].

Multiple connections with stress response were reported for TA systems in bacteria [3,52]. Various environmental factors including nutritional and genotoxic stresses trigger bacterial type I TA gene expression in *B. subtilis, S. aureus*, and *Escherichia coli* [6,40,47,48,53,54,55]. Several SOS-inducible type I TA systems have been identified in *E. coli,* e.g., *dinQ-agrB*, *tisB-istR-1*, and *symE-symR* [54,55,56]. *B. subtilis* BsrG-SR4 represents the first temperature-dependent type I TA [47,48]. In a multistress-responsive type I TA system, *bsrE*-SR5 from *B. subtilis* and *bsrE* mRNA is affected by temperature shock and alkaline stress, and the antitoxin RNA SR5 amount is influenced by pH, anoxia, and iron limitation stresses, with the last response being dependent on the alternative Sigma B factor [50]. In relation to the stress response in *B. subtilis*, putative binding sites of ResD transcription regulator induced upon oxygen stress have been identified upstream of the promoter regions of *bsrG, bsrH*, and *bsrE* type I toxin genes [43]. We have recently provided new data on the co-regulation of type I TA and CRISPR-Cas systems via the general stress response Sigma B factor in *C. difficile* that could be relevant for responses to stresses encountered by this pathogen inside the host [23]. In *C. difficile*, the Sigma B-dependent promoters could be identified in the regulatory regions of the majority of identified type I TA loci for both toxin and antitoxin genes [23,24] (Table 1, Figure 3). Interestingly, the MazEF type II TA module is encoded within the *sigB* operon in *S. aureus* with possible regulatory connections [57].

In the human pathogen *S. aureus*, several type I TA modules have been identified within pathogenicity islands (*sprA1*-SprA1AS, *sprA2*-SprA2AS, *sprG1-*SprF1) and the core genome (*sprG2*-SprF2, *sprG3*-SprF3, *sprG4*-SprF4), raising the question on the possible cross-regulation between homologous systems and their redundancy [53,58,59,60]. In addition, in the *S. aureus* Newman strain, an unusual condensed sRNA cluster could contain a novel type I TA system with the *srn_9343* gene, encoding a secreted peptide that presents sequence similarities to RelE type II toxin and Srn_9344 *cis*-antisense RNA that could function as an antitoxin [61]. As for other bacterial TA systems, *S. aureus* type I TA modules were related to stress adaptation with specific functions that could be suggested since they respond to different stress factors, including osmotic shock, nutrient starvation, and oxidative and acidic stresses [40,53,59]. The analysis of the possible crosstalk between several copies of homologous type I TA systems also suggested that each TA module might have specific roles. For example, the toxic peptides associated to SprA systems exhibit different features, PepA1 having both antimicrobial and hemolytic activity and PepA2 being mostly cytotoxic [40,53,60,62]. The SprG1-SprF1 system produces two toxic SprG1 peptides that can be secreted and lead to lysis of both human cells and competing bacteria [58], while core genome SprG-SprF TA copies would probably be involved in persistence associated with bacteriostatic toxin action [59]. As observed also for type I TA in *C. difficile*, each SprF antitoxin specifically neutralizes its cognate SprG toxin; however, heterologous cross-regulations between the RNA from non-cognate pairs were also observed. Similarly, only specific action in *trans* of *cis*-encoded antitoxin to prevent the translation of its cognate toxin was demonstrated for SprA modules [53].

A coordinated regulatory crosstalk has been recently described between type I *txpA*-RatA TA system and adjacent *mazEF* type II TA system in another human pathogen *Enterococcus faecalis* [63]. In addition to the type II TA autoregulation, the MazEF is also involved in the activation of type I antitoxin *ratA* transcription, where RatA in turn controls the type I toxin mRNA *txpA* levels affecting its stability.

## 4. TA Regulation

Two major mechanisms involved in the regulation of toxin expression and RNA decay within type I TA systems have been defined [6,42,46,49,53,56,60,64]. Antitoxin can affect the toxin mRNA translation or mRNA degradation, or do both as described for dual-acting SR4 antitoxin in *B. subtilis* [46]. In *C. difficile,* the RNA antitoxins from type I TA systems shared a long complementary region with convergently transcribed toxin mRNA suggesting an RNA degradation mechanism for antitoxin action [23,24]. The majority of bacterial type I antitoxins function through inhibition of toxin mRNA translation. They can either base pair with a ribosome-binding site or interfere with other translational elements to control toxin expression. mRNA folding would be also important for the control of type I toxin expression, as recently shown for TisB, ZorO, and DinQ in *E. coli* and for AapA1 in *Helicobacter pylori* [35,64,65,66,67]. The presence of a long 5′UTR (untranslated region) in type I toxin mRNA was highlighted as a target of antitoxin action [56,64]. Such long 5′UTR regions were also observed in type I toxin mRNA in *C. difficile* and could be important for the regulatory processes [23]. Among additional mechanisms to prevent toxin expression could be cited the sequestration of the RBS (ribosome-binding site) of toxin mRNA within a stable secondary structure and the requirement of a processing event to convert the toxin mRNA to a translationally active form [6,43,56]. Even if antitoxin RNA acts mainly through translational inhibition of toxin mRNA, the TA RNA duplex could be the target of degradation by RNase III. For example, this duplex-specific endoribonuclease was shown to cleave double-stranded RNA regions formed through base-pairing interactions between antitoxin RNA and complementary toxin mRNA for *txpA*-RatA system within the *B. subtilis skin* element [31,42]. The protection against the prophage-encoded toxin expression through antitoxin RNA was demonstrated to explain the essential role of RNase III in this bacterium [42]. In addition to RNase III, other ribonucleases could also contribute to the toxin and antitoxin RNA decay including RNase E in *E. coli* [68] and RNase Y and RNase J in *B. subtilis* [6,43,50].

In accordance with the data for other bacteria, the measurements of transcript half-lives showed that type I TA modules in *C. difficile* produce a rather stable toxin mRNA (estimated half-life of 36 min for *CD2907.1/CD0956.2* and 89 min for *CD0977.1*) and a less stable antitoxin RNA (estimated half-life of 13 min for major 125-nt transcript of RCd9/RCd10 and 8 min for major 150-nt transcript of RCd11) [23,24]. The analysis of the effect of RNase depletion on the stability of TA transcripts suggests the potential implication for the RNase J and RNase Y and, to a lesser extent, RNase III in the degradation of toxin mRNA. For antitoxin RNA the role for the RNase Y in the antitoxin RNA degradation could be also suggested [23,24]. For RCd9-*CD2907.1* and RCd10-*CD0956.2* TA pairs, the stable duplex formation was reported in vitro and these full TA duplexes could serve as a substrate for the efficient degradation by *E. coli* RNase III [23].

The RNA chaperone protein Hfq is required for *trans*-encoded sRNA action supporting their interactions with mRNA targets in Gram-negative bacteria [69]. Since the antisense RNA antitoxins share perfect complementarity with toxin mRNAs, the need for the RNA chaperone would not be generally expected for type I antitoxin action. Interestingly, the depletion of Hfq resulted in a moderate destabilization of *CD0977.1* toxin mRNA and antitoxin RCd11 RNA, and in a slight decrease in antitoxin RCd9/RCd10 half-life in *C. difficile* [23,24]. Currently, only one type I TA system *ralR*-RalA in *E. coli* requires Hfq for antitoxin function that appears to stabilize RalA [6,70]. No need for Hfq for an antitoxin control mechanism was reported in *B. subtilis* [43]. However, TA interaction regions were associated with Hfq in co-immunoprecipitation experiment [71]. All newly identified TA transcripts in *C. difficile* were also enriched in an RNA sample associated with Hfq in a co-immunoprecipitation assay (unpublished results, [72]) suggesting that Hfq might contribute in some way to the regulatory mechanisms involved in TA control.

## 5. Potential Functions of Type I TA Systems

Despite the continuous efforts on the study of TA systems, their biological role remains unknown in most cases. Among multiple functions discussed in the literature, only three have received experimental validation. This includes the stabilization of mobile genetic elements, abortive phage infection, and persister cell formation [1].

Initially, TA systems were shown to be important for the maintenance of plasmids through post-segregation killing mechanism [2,5]. So far, the role of numerous chromosomal TA modules remains largely enigmatic, but similar to our findings for *C. difficile* [24], a possible implication in the stabilization of chromosomal regions could be emphasized, for example, for type I TA in *B. subtilis* [43]. In *V. cholerae*, a proteinous TA module *mosAT* promotes the maintenance of an integrative conjugative element STX conferring resistance to multiple antibiotics [73], while in *Shewanella oneidensis,* a type II TA system ParE_SO_-CopA_SO_ stabilizes the prophage CP4So [74]. Another example underlines the role of chromosomal type II TA modules in the stabilization of massive superintegron arrays [75]. In *C. difficile*, as in other bacterial pathogens, the role of prophages in the physiology and virulence is being actively discussed [76,77]. It has been recently demonstrated that prophages influence toxin production suggesting that their maintenance could be critical for *C. difficile* pathogenesis [78,79].

In addition to the stabilization of genomic regions, other functions could be suggested for chromosomal TA modules. Among other mechanisms, abortive infection provides the resistance to bacteriophage infection to the bacterial population through the altruistic suicide of infected cells induced by TA module activation [1,7]. Abortive infection capacity has been demonstrated mainly for type III and type IV TA modules [7,20,80], but the implication for phage resistance for several type I and type II TA systems was also reported [81,82,83]. The recently identified co-localization of type I TA modules and CRISPR arrays in *C. difficile* chromosome suggests potential functional link between these systems for the defence against bacteriophage infection [23]. In relation to phage defence, the massive activation of archaeal defence genes, including induction of CRISPR and TA during viral infection, was recently reported [84].

Bacterial TA systems, including type I TA, were shown to influence persister cell and biofilm formation, as well as general stress response [52]. Persister cells are defined as a bacterial subpopulation achieving a transitory phenotypic conversion to a dormant state associated with tolerance to antibiotics and other stresses [1,5]. The roles of type II and type I TA modules, including TisB-IstR-1 in *E. coli* in the formation of persisters induced under stress conditions, have been suggested [4,66,85,86,87,88]. The first functional chromosomal type I TA system in Streptococci has been described in oral pathogen *Streptococcus mutans* and the role in persister cell formation has been suggested for this Fst-Sm-SRSm system [89]. One hypothesis to explain the potential role of stress-induced type I TA modules in metabolic and stress adaptation in *B. subtilis* is the bacteriostasis induction helping dormant cells to cope with stresses [6,43]. A recently described type I TA-like system *grtABC*-AsgR is involved in the stress response in *Corynebacterium glutamicum* [90]. In *C. difficile*, the identification of Sigma B-dependent promoters upstream of type I toxin and antitoxin genes also suggests their potential role in stress response mechanisms in this pathogen [23,24].

The induction of TA modules and their potential bactericidal action within biofilm communities have been discussed [52,91]. For example, the role of TxpA type I toxin from the *skin* element has been suggested regarding the elimination of defective cells to preserve symmetry in *B. subtilis* biofilms [92]. In *C. difficile*, we have shown that type I TA systems are induced in biofilm conditions suggesting the control of these systems via community-behaviour associated factors [23].

Increasing evidence highlights the key roles of TA systems specifically in bacterial pathogens [91,93,94]. One recent study reveals that *Salmonella enterica* serovar Typhimurium uses distinct type I and type II TA modules to regulate its intracellular lifestyle [94]. The increased number of TA modules was observed in intracellular bacterial pathogens further emphasizing their implication in virulence repertoire. Comparative genomic study showed that the genomes of most dangerous epidemic bacteria are characterized by the accumulation of TA modules [93]. TA systems could also directly contribute to the bacterial antibiotic resistance and pathogenicity via stabilization of pathogenicity islands, through the lysis of host cells, or by regulating the expression of virulence factors [91]. For example, similarly to findings in *S. aureus*, a selective endoribonuclease activity affecting virulence factor mRNA stability has been suggested for type II TA toxin MazF in *C. difficile* [21].

## 6. Possible Applications of Type I TA Systems

Unique features of bacterial TA modules provide a great potential for development of new biotechnological and therapeutic applications including genome editing, plasmid maintenance tools, selective reporter genes, new antimicrobials, and eukaryotic cell killing agents [45,95,96,97,98].

Several efficient strategies have been already described to reprogram the TA systems for the bacterial genome editing based essentially on the type II TA systems [96,99]. For example, type II RelBE TA was used to develop the toxin counter-selectable cassette regulated using an antitoxin switch for a wide variety of genetic modifications in Gram-positive bacteria including large-scale deletions and insertions, and gene knockdown and replacement, as well as point mutations [100]. In addition, a well-characterized *B. subtilis* type I TA module *bsrG*-SR4 has been recently used to design a new antitoxin RNA-guided gene repression system in this model bacterium [101].

In *C. difficile*, despite the continuous efforts, the efficient genetic tools are still needed to accelerate the research on this major enteropathogen. Based on our recent characterization of a functional *CD2517.1*-RCd8 type I TA module in *C. difficile* [23], we have developed a new genetic tool to improve the efficiency of the existing allelic exchange mutagenesis system in this bacterium [24]. Inducible type I toxin expression serves as an efficient counter-selection marker for isolation of deletion mutants in *C. difficile*.

Accumulating knowledge on TA modules from pathogenic bacteria led to the innovative strategies for design of new antimicrobials specifically targeting these pathogens [45,95,96,97,98]. The drugability of TA systems was the focus of several recent reviews that mostly discussed the type II and type III TA modules [95,97,98]. Among suggested strategies to make use of the toxin for pathogen self-targeting resides the triggering of the activation of TA systems by external signals, the inhibition of antitoxin synthesis, the activation of host enzymes degrading the antitoxin, or the design of specific inhibitors to disrupt the toxin-antitoxin interactions [95,97]. The specificity of drug delivery and action together with the toxicity to commensal microflora and to eukaryotic cells could be cited among important challenges for these therapeutic developments. The possibility to use recombinant bacteriophages is discussed to specifically deliver the toxin gene into the targeted pathogens. The toxin molecule itself could be considered as an antimicrobial candidate. For example, the type I PepA1 toxin from *S. aureus* can trigger cell lysis of both bacterial and human cells and was optimized via chemical modifications to increase the antibacterial potential and its stability, and to reduce the human cell toxicity [40,62]. Another type I toxin DinQ from *E. coli* has been considered as a potential anti-cell-envelope antibiotic against pathogenic *E. coli* infections [102]. The identification of functional type I TA modules broadly distributed in *C. difficile* strains opens promising perspectives on their use for development of alternative or synergistic strategies against the *C. difficile* infections.

## 7. Conclusions

Despites recent efforts, multiple facets of bacterial TA systems remain to be unveiled. In particular, the roles of chromosomal TA modules found in great numbers in different bacterial species need further investigations. In bacterial pathogens, this increasing knowledge could lead to better understanding of their stress management and adaptation strategies for the development of a coordinated pathogenesis program. Among clostridial pathogens, *C. difficile* attracted the attention of the scientific community due to the increased incidence and severity of infections and high rate of recurrences. The recent identification of more than ten type I TA modules distributed throughout its genome suggests their potential contribution to the success of this emerging human enteropathogen. Similar to other bacterial TA modules, the role of *C. difficile* type I TA in the stress response, biofilm structure, and genomic region stabilization could be hypothesized. As observed in well-studied Gram-positive bacteria, i.e., *B. subtilis* and *S. aureus*, many of these newly identified type I TA modules are located within prophages and prophage-like regions in the *C. difficile* chromosome. The intriguing association of some of them with CRISPR arrays related to defence capacities against foreign DNA elements raised the question regarding the potential functional link between these systems. The abundant chromosomal type I TA modules could thus help bacteria to keep the important genomic regions, including prophages that could shape the expression of virulence determinants and the adaptation capacities inside the host. Further studies will define the exact functions of these enigmatic chromosomal type I TA modules and specify the molecular mechanisms of their action and regulations involved. For the moment, most of the available information on the type I toxins is based on the overexpression experiments validating their toxic nature at high levels. However, their role at endogenous physiological levels remains unclear. The specific induction of small toxic proteins from type I TA modules could promote a reversible decrease in growth rate and help cells to survive under various stressful conditions. This could happen in the large proportion of cells or could be restricted to only limited bacterial populations, as suggested during the induction of persister formation. Depending on the level of toxin induction, the bactericidal function could be also considered, for example, for the elimination of defective cells from biofilm.

The small size of toxins and unique properties of type I TA systems make them potential interesting tools for numerous applications. In *C. difficile*, the inducible toxicity of type I toxins has been already successfully used for the elimination of plasmid-harbouring cells during the genome editing procedure. Further development of promising antimicrobial strategies based on TA systems could be considered, for example, by interfering with the TA component balance in favor of toxin expression. Future studies will provide new insights into the functioning of type I TA systems essential for further biotechnological and therapeutic applications in major pathogens.

## Figures and Tables

**Table 1:** Type I toxin-antitoxin systems identified in *C. difficile.*

Number	Toxin, Length ^1^	Antitoxin RNA	Location	Association	Comment ^2^	Ref.
1 *	CD0440.1, 46AA	CD630_n00150				
2 *^,3^	CD0904.1 (CD630_n00350), 35AA	AS *CD0904.1*	phiCD630-1		Prophage stabilization	[24]
3 *^,3^	CD0956.2, 53AA ^5^	RCd10	phiCD630-1	CRISPR 3/4	Prophage stabilization	[23,24]
4	CD0956.3, 34AA ^6^	AS *CD0956.3*	phiCD630-1		Prophage stabilization	[24]
5 *^,3^	CD0977.1, 47AA ^7^	RCd11	phiCD630-1	cdi1_5	Prophage stabilization	[24]
6 *	CD1233.1, 50AA	SQ808	skin	CRISPR 6		[23]
7 *	CD1418.2, 50AA	CD630_n00500		CRISPR 7		[23]
8 ^4^	CD1663.2, 59AA	CD630_n00610		CRISPR 9		[23]
9 *	CD2299.1, 50AA	SQ1641		CRISPR 11		[23]
10 *^,3^	CD2517.1, 52AA	RCd8		CRISPR 12		[23]
11 *^,3^	CD2889, 47AA ^7^	RCd12	phiCD630-2	cdi1_4	Prophage stabilization	[24]
12 *^,3^	CD2907.1, 53AA ^5^	RCd9	phiCD630-2	CRISPR 16/15	Prophage stabilization	[23,24]
13	CD2907.2, 34AA ^6^	AS *CD2907.2*	phiCD630-2		Prophage stabilization	[24]

^1^ The name and the length in amino acid (AA) of small toxic protein are provided. ^2^ The proposed function of the TA pair is indicated. The location within the prophage and prophage-like regions, as well as those associated with CRISPR arrays or c-di-GMP-responsive riboswitches, are pointed out. ^3^ Detailed analysis of these TA pairs was provided. *CD0904.1* gene was previously annotated as ncRNA gene CD630_n00350. ^4^ A divergent sequence for a small protein associated with an antisense RNA. ^5,6^ The 100% identical proteins within homologous phiCD630-1 and phiCD630-2 prophage regions of 53 and 34 amino acids in length, respectively. ^7^ The 95.7% identity proteins within homologous prophage regions. * For these TA modules, in addition to Sigma A-dependent promoter, a Sigma B-dependent promoter was identified upstream of the toxin and antitoxin genes. “AS” means antisense RNA antitoxin. Other names of antitoxin RNAs are given according to previous publications [23,26].
